# VLC-Based Positioning System for an Indoor Environment Using an Image Sensor and an Accelerometer Sensor

**DOI:** 10.3390/s16060783

**Published:** 2016-05-28

**Authors:** Phat Huynh, Myungsik Yoo

**Affiliations:** School of Electrical Engineering, Soongsil University, Dongjak Gu, Seoul 06978, Korea; phathuynh@ssu.ac.kr

**Keywords:** indoor positioning, visible light communication, LED, image sensor, accelerometer sensor, photodiode (PD)

## Abstract

Recently, it is believed that lighting and communication technologies are being replaced by high power LEDs, which are core parts of the visible light communication (VLC) system. In this paper, by taking advantages of VLC, we propose a novel design for an indoor positioning system using LEDs, an image sensor (IS) and an accelerometer sensor (AS) from mobile devices. The proposed algorithm, which provides a high precision indoor position, consists of four LEDs mounted on the ceiling transmitting their own three-dimensional (3D) world coordinates and an IS at an unknown position receiving and demodulating the signals. Based on the 3D world coordinates and the 2D image coordinate of LEDs, the position of the mobile device is determined. Compared to existing algorithms, the proposed algorithm only requires one IS. In addition, by using an AS, the mobile device is allowed to have arbitrary orientation. Last but not least, a mechanism for reducing the image sensor noise is proposed to further improve the accuracy of the positioning algorithm. A simulation is conducted to verify the performance of the proposed algorithm.

## 1. Introduction

During the last two decades, researchers have been trying to develop positioning techniques using VLC to provide precise location information. As it turns out, currently, VLC is able to support the indoor environment positioning function and is gradually starting to take control over the previous generation of positioning techniques using global positioning system (GPS), infrared (IR), radio frequency identification (RFID), bluetooth, wireless local area network (WLAN), ultrasound, *etc*. The main reasons that make VLC favorable for positioning are not only its capability to overcome most of the common obstacles with which the other techniques face, but also because it is friendly to human health and has efficient power consumption [1]. For example, GPS has been extensively used for positioning in outdoor positioning such as in car navigation, mobile phone, *etc*. However, for indoor positioning, GPS has shown poor performance since most of the GPS signals from satellites are blocked by the walls [2]. Other techniques for indoor positioning using WLAN, RFID, *etc.* have problems due to the system instability, long response time, and low accuracy [3,4,5]. Therefore, recently, VLC has gained significant attention from researchers for developing positioning systems.

The authors of [6] introduced VLC communication and positioning techniques from fundamental to advanced levels. An in-depth survey on VLC based positioning technology was conducted in [7]. Basically, VLC positioning techniques can be classified into two types: PD-based or IS-based system. The PD-based indoor positioning application has been widely developed because of the high sensitivity of PD to the light [8,9,10,11]. Although PDs are inexpensive, most hand-held devices are not equipped with PDs but cameras. Therefore, using PD for positioning would require additional cost compared to using IS. Another main advantage of IS compared to PD is that IS can spatially separate light sources. As a consequence, positioning systems using IS do not require multiplexing techniques, and the positioning accuracy would not be affected by ambient light noise as well as a multipath problem. For these reasons, the IS-based positioning technique has become a strong candidate for the next generation of indoor positioning systems.

Existing works on IS-based positioning algorithms can be found in [12,13,14,15]. In general, these methods used IS to capture images of LEDs. Then, position of the IS is determined based on the relationship between the known 3D coordinates of LEDs in the real world and the 2D coordinates of LEDs in the image. These methods differ from each other through deriving the position of the IS from available data. In [12,13], the collinearity condition was used to determine the position. While [12] used a normal lens attached to the IS, [13] used a fish-eye lens to achieve a much wider angle of view. However, the accuracy of [13] suffers from a lens distortion effect, and complicated calibration techniques must be applied to compensate for that effect. Instead of using the collinearity condition, which determines the position of the IS directly from the 3D world coordinates and 2D image coordinates of LEDs within one step, the authors of [14,15] find the position of the IS through two clear steps. Firstly, the distances from the IS to LEDs were calculated based on the 2D image coordinates of LEDs. Then, from these calculated distances and the 3D world coordinate of LEDs, a set of quadratic equations was formed. The position of the IS was determined by solving this set of equations. This algorithm was firstly proposed in [13] and later implemented in [15]. Compared to the collinearity method, it has clearer representation and implementation. However, it also has two major drawbacks. First, this algorithm requires two image sensors for determining the distance from the IS to LEDs in the first step. Second, this algorithm was built based on the assumption that the IS are parallel to the ceiling where the LEDs are installed. Obviously, this assumption would be impractical in many applications because a hand-held device can be tilted at any angle.

In this paper, a positioning algorithm is proposed to overcome the limitations of existing algorithms mentioned above. Our proposed algorithm determines the position of the IS through two clear steps. In the first step, the distances from the IS to LEDs are determined. However, instead of using two image sensors, the proposed algorithm just needs a single IS for determining the distances. In the second step, these distances are used to form a set of equations which will be solved to find the position of the IS. Another improvement in the proposed algorithm is that by using information from acceleration sensor, the IS is allowed to be tilted at any angle. In general, every image suffers from the image sensor noise at some levels. A high level of image sensor noise might severely degrade the positioning accuracy of the algorithm. However, the effect of image sensor noise is ignored in all existing positioning algorithms using IS. In this paper, a technique for reducing the image sensor noise is proposed to improve the accuracy of the positioning algorithm.

## 2. Proposed System

The proposed algorithm is divided into two separate phases. The first phase consists of discussing the physical design of the system and the geometrical relations between the IS and the LED panel under some crucial constraints. The second phase is devoted to explaining the cooperation with the AS and the construction of the homography view.

### 2.1. System Design and Estimation Algorithm

**Figure 1:**
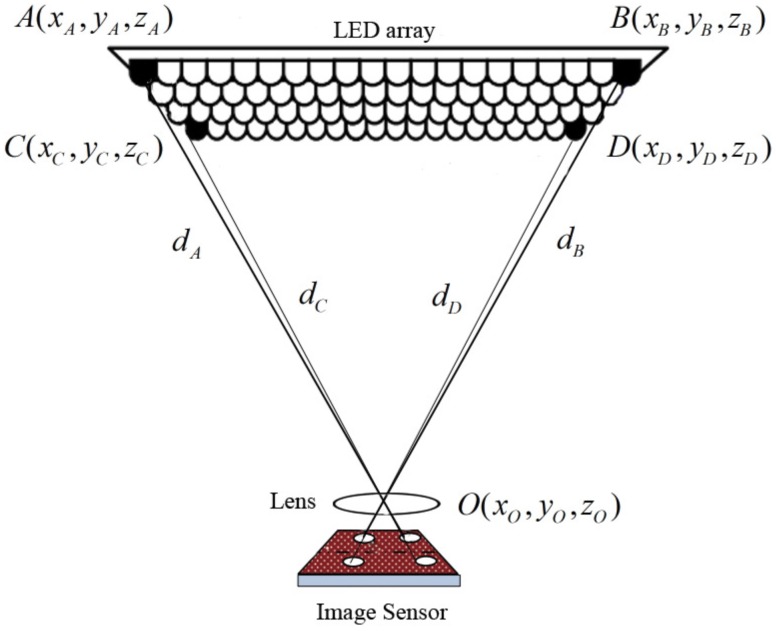
Proposed VLC-based positioning system.

We firstly introduce the environment where our proposed system could possibly be applied. This system is designed for an indoor environment, for instance, a room or an office. A system of LED panels is hung face-down on the ceiling. The number of LED panels depends on the size of the room so that the light from LED panels will cover the whole area. Each LED panel contains 2-dimensional array of LEDs. Figure 1 shows a demonstration for an LED panel. In this system, we use LEDs for illumination as well as for transmitting the information. At least four LEDs in an LED panel at a predetermined position on the ceiling transmit their three-dimensional coordinate information, which is received and demodulated by the IS at the unknown position. Authors in [16] have pointed out that LED intensity is assumed to be in the range of 4000 to 16,000 cd/m^2^, while that of the surrounding sources are around 400 lux for the indoor case. Therefore, once the images are captured, the LEDs will stand out from other sources. Hence, LEDs can be detected without difficulties. The IS is used for receiving the light signals and consists of a two-dimensional array of photosensitive elements. Each element or pixel acts as an individual photo sensor; hence, multiple LED signals can be detected and demodulated simultaneously by the IS. The IS receives the intensity modulated light from four LEDs through the optical lens and demodulates the 3D coordinate information of LEDs. Authors in [17,18] proposed an advanced technique to demodulate information that was captured by the image sensor.

For simplicity, we relax the complex conditions on the direction of the camera by assuming that the camera is facing directly to the ceiling and the two-dimensional IS space is parallel to the ceiling plane. This assumption is critical because it makes the algorithm less complicated, more accurate and reduces the response time of the whole system. The other case where the camera is in the arbitrary pose will be solved later with the accelerometer measurement.

Based on the predetermined coordinate system, such as the real world coordinate system of the ceiling, we denote *A*, *B*, *C* and *D* as four LEDs which transmit their 3D world coordinates, $\left(x_{A},y_{A},z_{A}\right)$, $\left(x_{B},y_{B},z_{B}\right)$, $\left(x_{C},y_{C},z_{C}\right)$ and $\left(x_{D},y_{D},z_{D}\right)$, respectively. These LEDs are randomly chosen among LEDs inside an LED panel. An illustration of the system’s physical arrangement is shown in Figure 1. The desired position of the camera is denoted as *O* and its world coordinate $\left(x_{O},y_{O},z_{O}\right)$ is the optical center of the lens. The unknown 3D world coordinate $O(x_{O},y_{O},z_{O})$ is estimated in this scheme. Furthermore, we denote the distances from the camera to each reference LED A,B,C,D as $d_{A},d_{B},d_{C},d_{D}$, respectively. These distances are calculated from the geometrical relationship of the image of LEDs in the IS.

**Figure 2:**
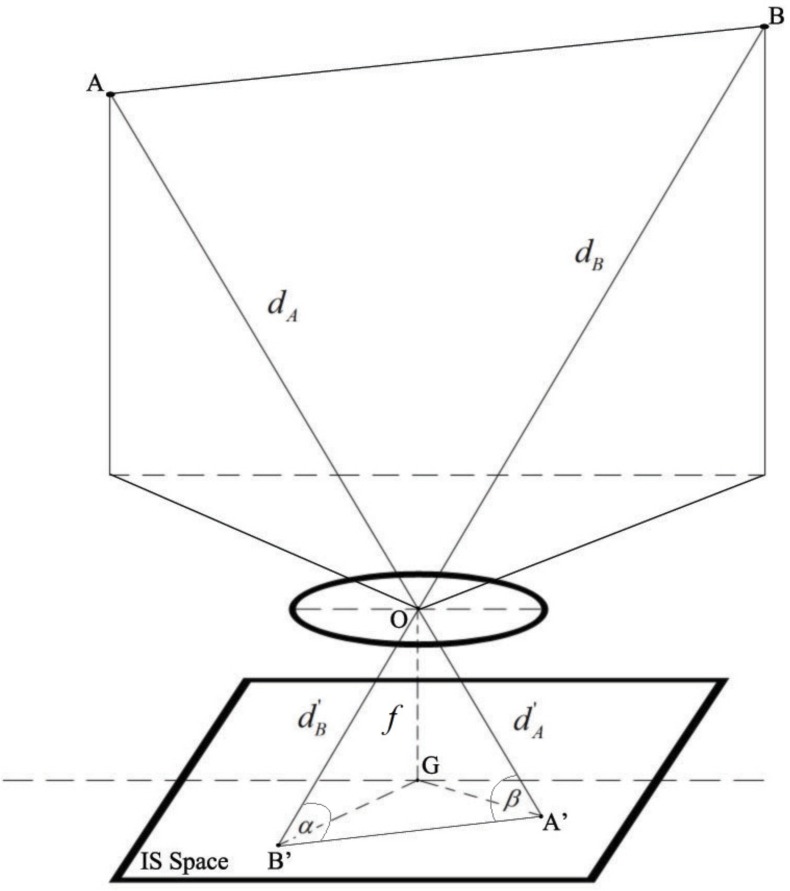
Positioning algorithm.

Figure 2 shows the geometrical relation of one pair of LEDs—for example, *A* and *B*. From each pair of LEDs, we can derive their distances to the camera. Based on the information regarding camera specifications, such as the focal length of the lens and the image sensor resolution size, the procedure for distance calculation is as follows.

We firstly detect the position of LED images in the 2D image coordinate. The accurate pixel is chosen simply by taking the pixel with the highest intensity among the pixels lit up by the LEDs. The advantage of this method and noise compensation are analyzed in the next section. Based on the fundamental calculus properties we obtain the following system of equations:

(1) $$\left\{\begin{array}{c} d_{A}^{{}^{\prime }2}=f^{2}+GA^{\prime 2}\\ d_{B}^{{}^{\prime }2}=f^{2}+GB^{\prime 2} \end{array}\right.$$

where *f* is the focal length of the camera lens, $A^{\prime }$, $B^{\prime }$ are the image of *A*,*B* on the IS, $d_{A}^{{}^{\prime }},d_{B}^{{}^{\prime }}$ are the distances from $A^{\prime },B^{\prime }$ to *O* as illustrated in Figure 2, and *G* is the center of the image sensor, and it is assumed that *G* is the origin of the 2D image coordinate. Then, according to the law of cosine, we obtain the following equations:

(2) $$\left\{\begin{array}{c} \alpha =\arccos\left\{\frac{A^{\prime }B^{\prime }+d_{B}^{{}^{\prime }}-d_{A}^{{}^{\prime }}}{2A^{\prime }B^{\prime }d_{B}^{{}^{\prime }}}\right\}\\ \beta =\arccos\left\{\frac{A^{\prime }B^{\prime }-d_{B}^{{}^{\prime }}+d_{A}^{{}^{\prime }}}{2A^{\prime }B^{\prime }d_{A}^{{}^{\prime }}}\right\} \end{array}\right.$$

where *α* and *β* are the angles formed from the LED’ light and the IS space through the pinhole camera model.

From the real world coordinate, since it is assumed that the focal length is known, GA and GB can be obtained through image processing. Then, the two angles *α* and *β* can be determined. Since the world coordinates of *A* and *B* are known, the distances $d_{A}$ and $d_{B}$ can be calculated using Equation (3):

(3) $$\left\{\begin{array}{c} d_{A}=\frac{\cos\beta \sin\alpha }{\sin(\alpha +\beta )\cos\alpha }AB\\ d_{B}=\frac{\sin\beta \cos\alpha }{\sin(\alpha +\beta )\cos\beta }AB \end{array}\right.$$

Similarly, for each pair of LEDs, we can obtain the distances $d_{A},d_{B},d_{C},d_{D}$ without any difficulty. Hence, we are able to form a set of four quadratic equations that can be solved to obtain the position *O* of the camera.

(4) $$\left\{\begin{array}{c} {\left(x_{O}-x_{A}\right)}^{2}+{\left(y_{O}-y_{A}\right)}^{2}+{\left(z_{O}-z_{A}\right)}^{2}=d_{A}^{2}\\ {\left(x_{O}-x_{B}\right)}^{2}+{\left(y_{O}-y_{B}\right)}^{2}+{\left(z_{O}-z_{B}\right)}^{2}=d_{B}^{2}\\ {\left(x_{O}-x_{C}\right)}^{2}+{\left(y_{O}-y_{C}\right)}^{2}+{\left(z_{O}-z_{C}\right)}^{2}=d_{C}^{2}\\ {\left(x_{O}-x_{D}\right)}^{2}+{\left(y_{O}-y_{D}\right)}^{2}+{\left(z_{O}-z_{D}\right)}^{2}=d_{D}^{2} \end{array}\right.$$

We use the least square algorithm to estimate the coordinate of *O*. Since there are only three unknown variables, the four equations in Equation (4) are sufficient to find $\left(x_{O},y_{O},z_{O}\right)$.

The condition to obtain a solution for the Equation (4) was identified in [14]. This condition might not be satisfied if three or more LEDs lie in the same line. In order to overcome this problem, we adopt an algorithm that was suggested in [14]. More specifically, to avoid the straight line alignment, a small value of each LED on only the *z*-axis is adjusted, and then the vector estimation is applied to obtain the precise result.

### 2.2. Accelerometer Sensing and Homography Construction

#### 2.2.1. Accelerometer Sensing

In the previous subsection, we solve the positioning problem in the simplest case where the IS is parallel to the ceiling. However, in practice the camera might have arbitrary orientations and the algorithm explained above would fail to determine the position of the camera. To overcome this problem, we suggest utilizing an AS, which is one of the core components in the most current handset devices, in order to obtain the knowledge related to the orientation of the camera. Then, the positioning algorithm will use this knowledge to determine the position of the camera at arbitrary orientations.

**Figure 3:**
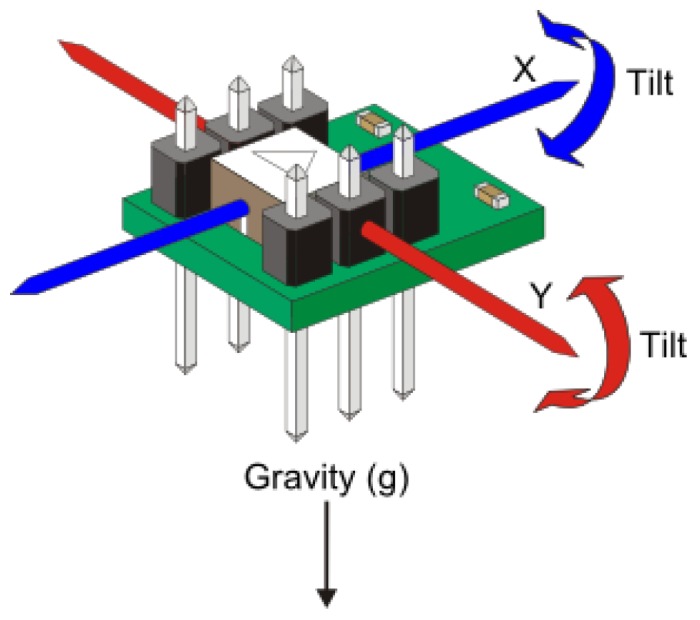
Accelemeter sensor.

The AS is an advanced, ultra-small sensor that aims for highly integrated, low-power consumer electronic applications. The accelerometer allows low-noise measurement of acceleration in three perpendicular axes, and thus senses tilt, motion, shock and vibration in smart phones, tablets, computer peripherals, human-machine interfaces, augmented reality devices, ubiquitous sensor networks, *etc*. Our proposed positioning scheme requires that the AS measures the tilt forward and backward on the *x*-axis, and it measures the tilt side to side on the *y*-axis. The AS’s outputs and specifications can be easily accessed and fully controlled via the application program interface (API) functions provided by the manufacturer. Figure 3 depicts an AS.

Under the real world coordinate system, we define the rotation matrix presenting the IS’s rotation of the current pose to the pose where the IS is parallel to the ceiling. Particularly, from the AS, the information about the tilt shifts on the *x*-, *y*- and *z*-axes will be extracted in order to form the rotation matrix. The AS is highly sensitive; hence, any minor changes in movement or motion are immediately collected and processed without affecting the time response of the entire system. In order to form the rotation matrix, let us consider that the *ψ*, *ξ*, *ζ* are the tilt differences measured from the AS corresponding to the *x*-, *y*-, *z*-axes, respectively. The rotation matrix can be obtained from three rotation matrix components using matrix multiplication:

(5) $$R=R_{x}\left(\psi \right)R_{y}\left(\xi \right)R_{z}\left(\zeta \right)$$

where $R_{x}\left(\psi \right)$, $R_{y}\left(\xi \right)$ and $R_{z}\left(\zeta \right)$ are the rotation related to the *x*-, *y*-, *z*-axis, respectively, and are in the form of

$$R_{x}\left(\psi \right)=\left[\begin{array}{ccc} 1 & 0 & 0\\ 0 & cos(\psi ) & sin(\psi )\\ 0 & -sin(\psi ) & cos(\psi ) \end{array}\right]R_{y}\left(\xi \right)=\left[\begin{array}{ccc} cos(\xi ) & 0 & -sin(\xi )\\ 0 & 1 & 0\\ sin(\xi ) & 0 & cos(\xi ) \end{array}\right]$$

$$R_{z}\left(\zeta \right)=\left[\begin{array}{ccc} cos(\zeta ) & sin(\zeta ) & 0\\ -sin(\zeta ) & cos(\zeta ) & 0\\ 0 & 0 & 1 \end{array}\right]$$

Hence, the rotation matrix takes the following form:

(6) $$R=\left[\begin{array}{ccc} C(\xi )C(\zeta ) & C(\xi )S(\zeta ) & -S(\xi )\\ -C(\psi )S(\zeta )+C(\zeta )S(\psi )S(\xi ) & C(\psi )C(\zeta )+S(\zeta )S(\psi )S(\xi ) & C(\zeta )S(\psi )\\ S(\psi )S(\zeta )+C(\psi )C(\xi )S(\zeta ) & -S(\psi )C(\zeta )+C(\psi )S(\xi )S(\zeta ) & C(\psi )C(\xi ) \end{array}\right]$$

#### 2.2.2. Homography Construction

Together with the rotation matrix, we now discuss a way to create a pose reconstruction from two views, which is termed homography. Homography is related to the images captured from different poses of the camera looking at the same planar surface. The following discussion provides the mathematical relationship of objects’ coordinates in the related images. In our system, the problem is how to identify the new pixel coordinates of the LED as if the image is taken in the transformed pose of the camera. Let us consider the case in which the camera takes the image of LEDs in the arbitrary pose. We then use the AS to adjust the image as if IS is facing parallel to the ceiling plane without performing any physical movement of the camera.

Let us assume that we have two poses of the camera denoted as $P_{1}$ and $P_{2}$. $P_{1}$ stands for the free-pose of the camera, $P_{2}$ stands for the pose in which the camera is parallel to the ceiling. The 3D camera coordinate is assumed to have its origin at the projection center of the camera. The 3D world coordinate is assumed to have its origin at the center of the ceiling of the room. The rotation and the translation of the camera are defined as $R\in R^{3\times 3}$ and $T\in R^{3}$, respectively. In this case, we simplify the translation operator because we do not need changes in location, but in the pose only. The camera captures a collection of static reference LEDs, and the number of LEDs is assumed to be equal to or greater than four to avoid the coplanar and noncollinear conditions.

A reference LED has 3D camera coordinate $l_{P_{1}}^{i}={\left[x_{P_{1}}^{i},y_{P_{1}}^{i},z_{P_{1}}^{i}\right]}^{T}$ $\in R^{3}$, where the upper index *i* stands for the order of LEDs in a captured collection of feature points, and the lower index indicates the current pose of the camera. The standard geometric relationships can be applied to the coordinate system to develop the following equation:

(7) $$\begin{array}{cc} l_{P_{2}}^{i} & =H_{P_{2}P_{1}}l_{P_{1}}^{i} \end{array}$$

where $H_{P_{2}P_{1}}=\left(R_{P_{2}P_{1}}+\frac{T_{P_{2}P_{1}}}{d_{P_{1}}}n_{P_{2}}^{T}\right)$ is transformation matrix from $P_{1}$ to $P_{2}$, and $n^{T}$ is the constant unit vector that is perpendicular to the ceiling plane, and $d_{P_{1}}$ is the constant distance between the ceiling plane and the XY plane of the 3D camera coordinates. Equation (7) explains how we can transform the location of LED in the captured image under the pose $P_{1}$, which has arbitrary orientation, to that of the pose $P_{2}$, which is parallel to the ceiling, through the rotation matrix and the translation matrix. Literally, when the LED location corresponding to the pose $P_{1}$ is derived, we can get the location of LED corresponding to the pose $P_{2}$ by rotating and shifting camera to the pose $P_{2}$. The 3D camera coordinate $l_{P_{2}}^{i}$ can be translated into the 2D image coordinate using standard projective geometry as $p_{P_{2}}^{i}={\left[x_{P_{2}}^{i},y_{P_{2}}^{i},1\right]}^{T}$. Using the pinhole camera model as described in [19], the projective coordinate of the reference points are transferred to

(8) $$p_{P_{2}}^{i}=Al_{P_{2}}^{i}$$

where A is the invertible, upper triangular camera calibration matrix defined as:

(9) $$A=\left[\begin{array}{ccc} m & -mcos\phi & u\\ 0 & \frac{n}{sin\phi } & v\\ 0 & 0 & 1 \end{array}\right]$$

where u,v∈R represent the pixel coordinates of the principal point *G* of the IS, which is defined as the intersection of the optical axis with the image space, m,n∈R stand for the scaling factor of the pixel dimensions, and ϕ∈R is the skew angle between the camera axis. Combining Equations (7) and (8), the Euclidean relationship in Equation (7) can be expressed as follows:

(10) $$p_{P_{2}}^{i}=AH_{P_{1}P_{2}}A^{-1}p_{P_{1}}^{i}$$

Equation (10) describes the pixel coordinates of two images with the movement of the camera represented by the rotation matrix and translation matrix. Then, we obtain the pixel coordinate of the LEDs in the image taken with the free-pose camera, and we are able to derive the pixel coordinates of LEDs as if the image is taken when the camera is facing parallel to the world reference plane.

The whole system is combined together to provide a valid solution for the distances as required in Equation (4).

### 2.3. IS Noise Evaluation

In this section, we provide and discuss a sensor noise model for the camera. As mentioned in the previous subsection, we have to identify the pixel coordinate of the LED in the 2D IS coordinate space by picking up the pixel with the highest intensity. However, noise is generated by multiple sources such as ambient light noise and thermal noise; hence, it could have a significant impact on positioning accuracy. The noise is modeled under the assumption that the image is captured and processed under the gray scale, which generates an 8-bit color image; hence, the range of displayed colors is [0,255].

Let us consider the pixel-noise model of the following form:

(11) c(i,j)=r(i,j)+δ(r(i,j))ϵ(i,j)

where $\left(i,j\right)\in N^{2}$ is the pixel coordinate in the 2D IS sensor domain, $c:N^{2}\to N$ is the captured (recorded) signal intensity, $r:N^{2}\to N$ is the ideal signal where there is no source of noise affecting the original signal, $\epsilon :N^{2}\to R$ is the zero-mean independent random noise with standard deviation equal to 1, and $\delta :R\to R^{+}$ is a function of *r* that stands for the standard deviation of the overall noise component. Considering the expectation of both sides in Equation (11) would give the received signal on the average presented by the signal in the ideal case where no noise occurs.

In our modeling, we have to determine the model for the overall noise term. An appropriate model related to the intensity of the pixels is the Skellam distribution model [20]. The Skellam distribution can be used to measure the intensity difference between pixels in the spatial domain, as well as in the temporal domain.

The idea behind this model is to consider the intensity of each pixel that follows the Poisson distribution at the fixed time *t*. The number of photons determines the intensity of a pixel. Two consecutive images with the same scene are captured, and then the noise term is considered as the intensity difference in each pixel of the images. Hence, we model the IS noise as the difference between two Poisson distributions. The difference between two Poisson random variables is defined as Skellam distribution. The probability mass function for the Skellam distribution is the function of *k*, which is expressed as follows:

(12) $$f\left(k;\mu_{1},\mu_{2}\right)=e^{-(\lambda_{1}+\lambda 2)}(\frac{\lambda_{1}}{\lambda_{2}}{)}^{\frac{k}{2}}I_{k}\left(2\sqrt{\lambda_{1}\lambda_{2}}\right)$$

where, $\lambda_{1}$ and , $\lambda_{2}$ are the expected values of two Poisson distributions, and $I_{k}\left(z\right)$ is the modified Bessel function of the first kind. Then, the Skellam parameter of intensity difference is expressed by using statistics of the Skellam distribution:

(13) $$\left\{\begin{array}{c} \mu_{S}=\lambda_{1}-\lambda_{2}\\ \sigma_{S}^{2}=\lambda_{1}+\lambda_{2} \end{array}\right.$$

**Figure 4:**
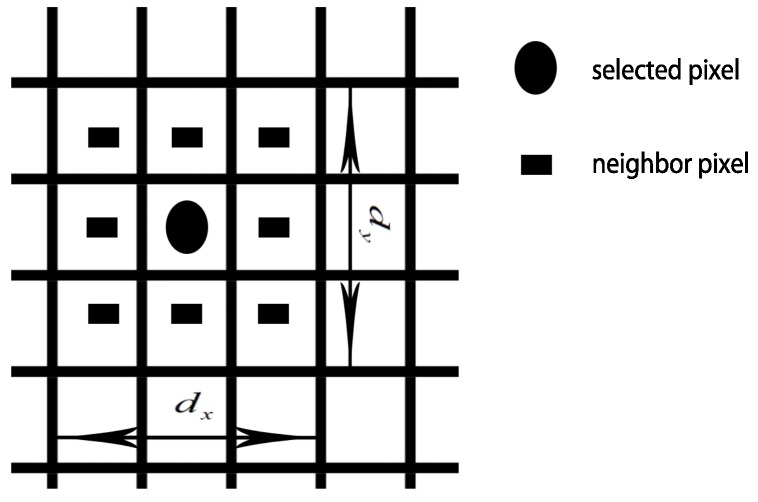
Neighbor pixel of the selected pixel.

In order to derive the noise factor, we have to estimate the parameters $\mu_{S}$ and $\sigma_{S}$ in Equation (13). For this estimation, we need at least two images from the static camera. However, in our proposed scenario, it is not possible to capture multiple images of the same scene; hence, to generalize our modeling, we should be able to estimate the noise from only a single image. By assuming that the noise components of each pixel in the spatial domain are mutually independent, which means that the noise distribution along the spatial domain is the same as that along the temporal domain, we can easily perform the estimation with only a single image. For each pixel on the captured image, we define the area of neighboring pixels, and the borders of this area are limited by $d_{x}$ and $d_{y}$ in Figure 4. The size of the neighboring area can be increased by expanding $d_{x}$, $d_{y}$. The estimation of the noise in each pixel can be done by subtracting the selected pixel intensity by the pixel intensities in the neighboring area, followed by taking the average of pixel intensity differences between the selected pixel and the pixel in the neighboring area to obtain the noise. Equation (14) is then applied to the spatial domain in order to derive the noise distribution’s parameters

(14) $$\left\{\begin{array}{c} \mu_{S}=\frac{\sum_{\left(d_{x},d_{y}\right)\in N^{2}}^{}c\left(i,j\right)-c\left(i+d_{x},j+d_{y}\right)}{n}\\ \sigma_{S}=\frac{\sum_{\left(d_{x},d_{y}\right)\in N^{2}}^{}{\left(\mu_{S}-c\left(i,j\right)-c\left(i+d_{x},j+d_{y}\right)\right)}^{2}}{n} \end{array}\right.$$

where $d_{x}$ and $d_{y}$ are the lengths of neighboring pixels in the horizontal and vertical directions, respectively, and *n* is the total number of pixels in neighbor area. Then, we can finally correct the estimated positioning result by subtracting the noise component from the recorded signal.

The above algorithm framework shows the pseudo code to estimate the parameters for the image sensor noise model and to adjust the value of the intensity of each pixel given by the Equations (11), (13) and (14).

## 3. Simulation and Discussion

### 3.1. Simulation Environment

The system performance is evaluated by Matlab. Firstly, it is important to discuss the field of view (FOV) concept, because once the IS is utilized as a reception device, the FOV is one of the core parameters of the system. The FOV is the extent of the observable world that can be seen at any given moment. In the case of optical instruments or sensors, it is a solid angle through which a detector is sensitive to electromagnetic radiation.

Basically, the wider the lens used, the larger camera works. The common built-in cameras of cell-phones are currently working with the length of focal lens raging from 18 mm to 22 mm, which provides quite wide FOV. Based on the given camera specifications such as the IS size, resolution and the length of focal lens, we are able to determine the FOV by the following equation:

(15) $$\phi =2arctan(\frac{w}{2f})$$

where *w* is the IS width and *f* is the length of the focal lens. Based on the popular cell-phone models in the market, we obtain the IS resolution typically ranging from 3267 to 4160 pixels in width and from 2448 to 3120 pixels in height, which produces an image of a size of 8 megapixels to 13 megapixels, respectively.

The image sensor size is around 4.8 × 3.6 mm${}^{2}$. According to Equation (15), FOV has values in the range of 24${}^{\circ }$ to 30${}^{\circ }$. Hence, the maximum working area is approximately 7 × 7 × 3.5 m${}^{3}$ with the constraint that the LED panel is mounted upon 3.5 m height of the ceiling. The conclusion regarding the working area size suggests that this proposed algorithm is sufficiently suitable for most indoor applications in which the room has the same shape of numbers. In addition to this, the tilt shift of the camera should not be more than 60${}^{\circ }$. In our simulation framework, we consider the range from 0${}^{\circ }$ to 60${}^{\circ }$ on both the *x*-axis and *y*-axis, respectively. Otherwise, significant degradation of FOV and working area size could cause a huge error in the algorithm accuracy. For some parts of the system performance evaluation, the IS resolution is fixed at a maximum size of 4000 pixels in width and 3000 pixels in height. The table of parameter summary is shown in Table 1.

### 3.2. Process for Simulating LED Images

To evaluate the accuracy of the proposed algorithm, we firstly need to simulate the LED images as if they are captured by a camera. Given the world coordinates of LEDs and the world coordinate and pose of the camera, the LED image can be simulated using a pinhole camera model.

**Figure 5:**
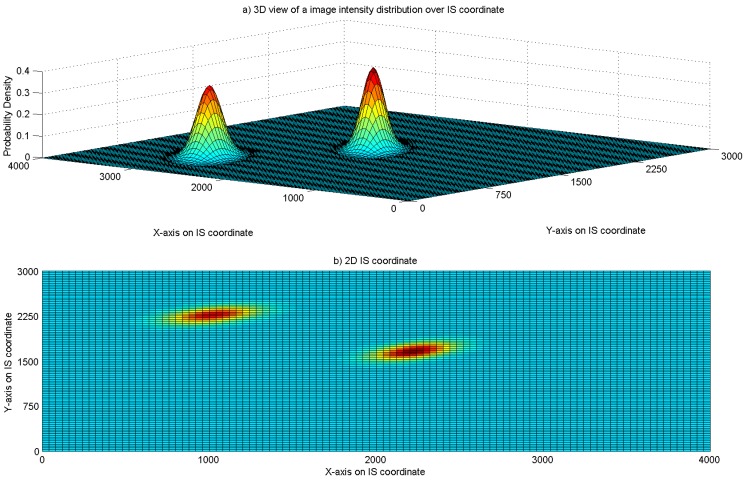
Pixel intensity distribution of a simulated image.

Figure 5 shows the simulated image of two LEDs having world coordinates of (5,15,35) and (3,19,35), respectively. The parameters of the camera and lens are presented in Table 1. The camera is assumed to have zero tilt angles. Figure 5a shows the intensity of the LED images. The bell shape of the 3D histogram reflects the assumption in the ideal case in which the electromagnetic radiation follows the rule of sight such that it comes directly from the LED with no interference from environmental noise. On the other hand, Figure 5b shows the 2D position of LEDs in the image. Through image processing, the pixel coordinates of the LEDs can be obtained. For this specific simulated image, noise reduction is not needed. However, in practice, fading propagation can also create some noise in the image which would potentially lead to an error in the positioning algorithm. We address these points in the later part of this section.

To evaluate the proposed algorithm, the camera is assumed to be placed at 350 × 350 points equally spaced in the room with certain poses. Each simulated point will be at a distance of 0.02 m from its neighboring points. Then, the images of LEDs that are taken by the camera at these positions and poses are simulated. After that, the proposed positioning algorithm is applied to these simulated images to identify the position of the camera. Finally, the positioning errors, which are the distance differences between the assumed positions and the estimated positions of the camera, are calculated. The average positioning error is calculated as the mean positioning error at these 350 × 350 position in the room.

### 3.3. Evaluation of the Effect of Camera Parameters on the Positioning Accuracy

#### 3.3.1. Effect of Sensor Resolution

**Figure 6:**
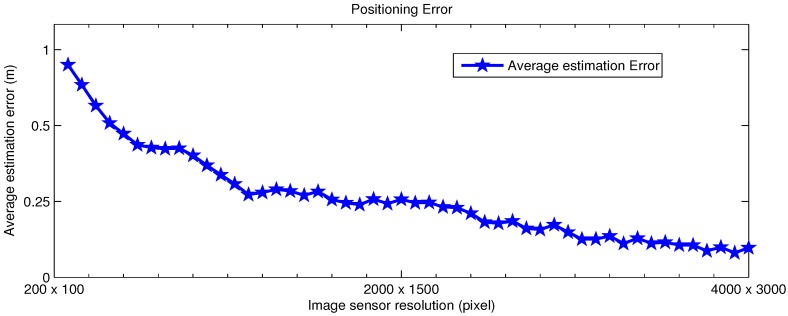
Estimation of error improvement along with the increase in IS resolution.

In order to show the effect of sensor resolution on the accuracy, the average positioning errors are calculated with the sensor resolution ranging from 200 × 100 to 4000 × 3000. The camera is assumed to have zero tilt angle, and the amount of noise is randomly added to the image with the uniform distribution between 0 dB and −9 dB. The other parameters are kept the same as in Table 1. The result is presented in Figure 6.

As shown in Figure 6, the average positioning error decreases when the sensor resolution increases, which is pretty much the expected result. The reason for this improvement in the positioning accuracy is that when the sensor resolution increases, the 2D image coordinates of LEDs obtained from the image are closer to their actual values.

It can also be seen that the positioning accuracy does not improve much after the sensor resolution exceeds the middle-high level of resolution of 2000 × 1500 pixels. This suggests that the proposed algorithm could be deployed in handsets which are not equipped with a good quality image sensor.

#### 3.3.2. Effect of AS Accuracy

In this part, we investigate the effect of AS accuracy on the positioning performance. We have been looking at a lot of accelerometers from various major manufactures and have done some basic simulation based on AS accuracy parameter to find out whether or not it has some critical impact on the system performance. Generally, accelerometer manufacturers produce their products reaching as high a level of accuracy as possible. Commercial accelerometer sensors typically take the error range of ±2 degrees during the normal operating conditions. For some applications, high accuracy accelerometer sensor can satisfy the requirement up to 0.5 degrees of error. Furthermore, researchers have shown some experimental results showing the error hits impressively at 1/1000 of a degree. In our case, we take AS errors ranging from 0.1 to 2 degrees on each axis for the analysis of positioning algorithm performance.

Table 2 shows small changes in positioning results regarding the estimated angle errors of the AS. Even when the angle errors are up to two degrees, the final positioning result does not change much compared to that without angle errors.

#### 3.3.3. Effect of Camera Tilting Angle

**Figure 7:**
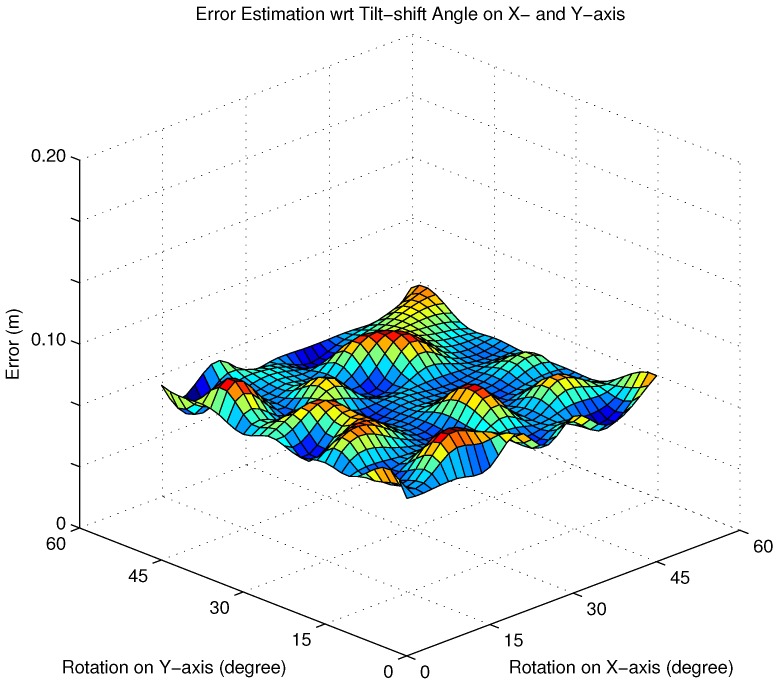
Estimated error with respect to the tilt angle.

To evaluate the effect of the camera tilt angle on the positioning accuracy, the average positioning errors are calculated with different poses of the camera. More specifically, the tilt angle on the *x*- and *y*-axes of the camera is set between $0^{\circ }$ to $60^{\circ }$. The camera is assumed to have the highest resolution of 4000 × 3000 pixels. According to the result in Section 3.3.2, we assume that AS has no effect on simulated results of this part and we use the same noise environment as in the previous simulation setup on this part. The other system parameters are kept the same as in Table 1. The simulation result is presented in Figure 7.

Taking advantage of the AS, regardless of pose of the camera, the proposed algorithm is able to identify the position of the camera. There is a slight difference in error estimation with different poses of camera. From Figure 7, it can be seen that the average positioning errors are always less than 0.1 m with all poses of the camera. The reason for the limit of the tilt angle of the camera is due to being based on some basic calculations in Equation (15). If the tilt angle exceeds $60^{\circ }$, the intersection of the FOV, corresponding to the IS pose, and the emitting area of the LED, are almost zero. At that time, the image sensor is unable to adequately capture the required reference LEDs on the ceiling, and hence, the position of the camera cannot be determined.

#### 3.3.4. Effect of Image Noise

Besides sensor resolution and tilt angle of the camera, image noise can also degrade the performance of the positioning algorithm. Image noise has been conventionally regarded as an additive Gaussian random variable. However, since the Gaussian noise model is not generally applicable, the Skellam distribution is introduced as a sensor noise model for the charge-coupled device (CCD) sensor and complementary metal-oxide semiconductor (CMOS) sensor as in [20], and it is also advanced for the visible light communication application in which only a single image is required.

**Figure 8:**
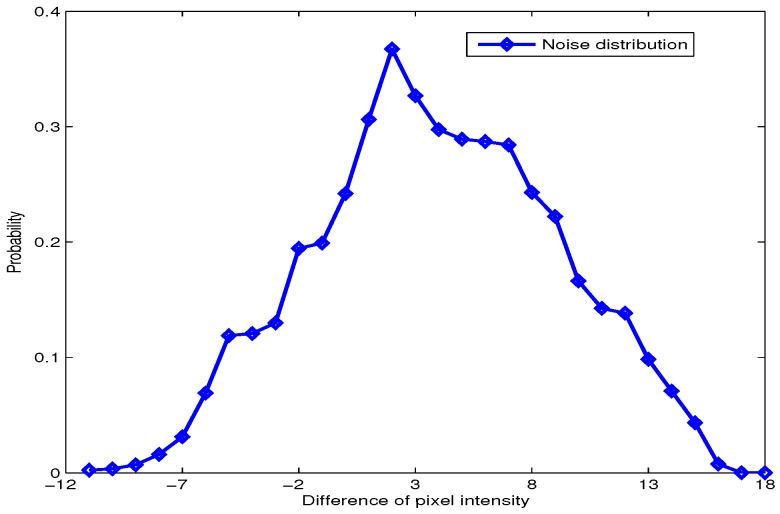
Histogram of noise.

In the simulation, Algorithm 1 is used to derive the noise, which is the difference in pixel intensity along the spatial domain. We first select the particular pixel to perform the calculation. The selected pixel is determined by choosing origin point *G* on a 2D image coordinate system as shown in Figure 2. We calculate pixel intensity differences between the the selected pixel and the pixels located in the neighbor area as explained in Figure 4. In our simulation setup, we set neighboring area’s parameters $d_{x}$ and $d_{y}$ equal to 11, which generates 120 intensity differences. Figure 8 shows the noise intensity distribution of the selected pixel over 120 pixels. Then, we estimate the noise distribution parameters by simply taking the average and standard deviation over the pixel intensity differences as in Equation (14). After assessing the amount of noise, represented by $\mu_{S}$ and $\sigma_{S}$, in the selected pixel, we adjust the selected pixel based on the noise parameters to obtain the meaningful information. We execute Algorithm 1 for every pixel to find the noise and reduce the error on the image-wide scale.

**Algorithm 1** Noise reduction algorithm  1: **procedure**
Noise Reduction Algorithm  2:     n←#ofpixels  3:     $\text{initialize}\;\mu_{S}\;\text{and}\;\sigma_{S}$  4:     initializeSSoID(Sumofsquareofintensitydifference)  5:     $\text{defind}\;\text{the}\;\text{neighboring}\;\text{area}\;d_{x},d_{y}$  6:     **for** each *i* ∈ #pixeloftheverticaldirection
**do**  7:         **for** each *j* ∈ #pixelofthehorizontaldirection
**do**  8:            **for** each pixelintheneighboringarea
**do**  9:                $\mu_{S}\leftarrow \mu_{S}+\frac{c\left[i,j\right]-c\left[i+d_{x},j+d_{y}\right]}{n}$  10:                $SSoID\leftarrow \frac{1}{n}{\left(c\left[i,j\right]-c\left[i+d_{x},j+d_{y}\right]\right)}^{2}$  11:                $\sigma_{S}=\mu_{s}-SSoID$  12:                $\text{Find}\;\lambda_{1}\;\text{and}\;\lambda_{2}\;\text{from}\;\mu_{s},\sigma_{S}$  13:            **end for**  14:         **end for**  15:     **end for**  16:     adjusttheintensityofeachpixel  17: **end procedure**

In order to show the effect of image noise on the positioning accuracy, we first add noises with different levels to the simulated image. Then, we apply our proposed noise reduction mechanism to these noise-added images, apply the positioning algorithm to the noise-reduced images, and then calculate the positioning errors.

**Figure 9:**
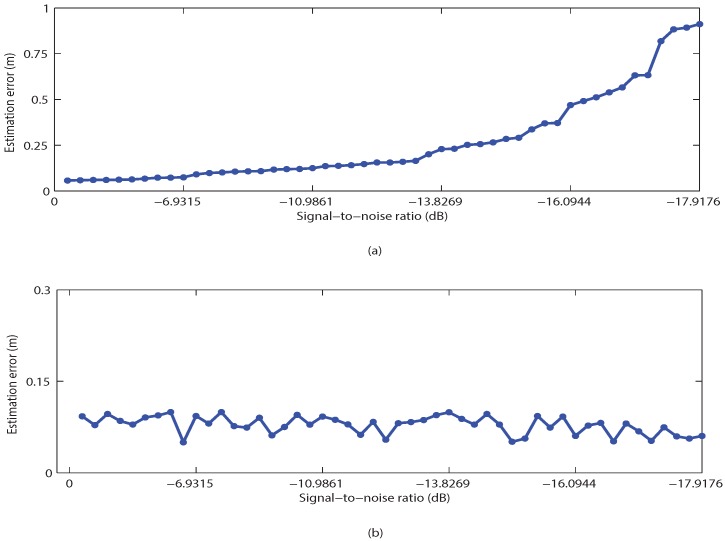
Noise elimination scheme performance. (**a**) Estimation errors of LED without noise elimination; (**b**) Estimation errors of LED with noise elimination.

Figure 9a shows the positioning accuracy of the proposed algorithm with varying levels of noise added to the image. It can be seen that when the level of noise increases, the positioning error dramatically increases. The effectiveness of the proposed noise reduction algorithm can be seen clearly in Figure 9b. From Figure 9, it can be seen that the positioning error is kept stable at all levels of noise added to the image.

### 3.4. Performance Comparison between Positioning Algorithms Using IS

The comparison on the performance of the proposed algorithm and existing positioning algorithms using IS is given in Table 3. It can be seen that, despite the simple mechanism, the proposed algorithm can achieve a good performance compared to its counterparts. In addition, the accuracies of existing algorithms were reported under the conditions of a low level of image sensor noise. When the level of noise in the image is high, the performance of other algorithms would be degraded while our proposed algorithm can maintain its accuracy thanks to the noise reduction mechanism.

### 3.5. Discussion

Basically, our proposed positioning system is the VLC based method using a camera as a receiver; hence, a camera is required to receive and demodulate the transmitted signal. According to the current state of research on the VLC system, use of a camera still has the problem of a low data transmission rate. Thus, our proposed system is applicable to stationary or slowly moving targets. This problem is completely resolved with high-speed camera equipment, but it is costly and is not currently available for handset devices.

The main reason for significant error in most positioning systems is noise terms, which are usually superficially considered. In our modeling, only the overall noise term is explored, which means that separate noise factors are not dealt with individually. Hence, it is possible that one can achieve a higher degree of accuracy if one goes deeply into the model and gets rid of the individual noise terms. We will investigate this point in our further work in which we will attempt to enhance the system efficiency.

The positioning accuracy also depends on the detection of LED in images. As long as the luminance of LED is sufficient, the LED can be detected easily from the captured image. In other words, as long as the luminance of LED is sufficient, the positioning error changes very little, if any, with the change in the LED luminance. Of course, if the LED luminance is not sufficient, the detection of LED in the capture image would be difficult, and the positioning accuracy would decrease. However, in this paper, we assume the LED has a typical luminance from 4000 to 16,000 cd/m${}^{2}$, which is far from sufficient for the LED detection. Therefore, in this paper, the positioning error is assumed to be independent from the LED luminance.

## 4. Conclusions

In this paper, we propose a new positioning system for an indoor environment specialized for handset devices using an image sensor and an AS in handset devices. The construction of the whole system is separately divided into two phases. The first phase consists of finding the valid solution to the unknown camera position based on the Euclidean geometric relationship, with the assumption that the IS coordinate space is parallel to the world reference plane. The second phase is devoted to reconstructing the image with the free-pose camera. The noise factor is also evaluated to obtain more accurate results. We provide the simulations to verify the proposed theoretical mechanism. The system has shown a high level of positioning accuracy, practicality, and benefits of device simplicity.

## Figures and Tables

**Table 1:** Simulation parameters.

Parameter	Value
IS dimension	4.8 × 3.6 mm${}^{2}$
Number of pixels	4000 × 3000 pixels
FOV	24${}^{\circ }$
Focal length of the lens	18 mm
Positioning Area	7 × 7 × 3.5 m${}^{3}$
Number of simulated points in the room	122,500 (350 × 350)
Number of LEDs	4
LED 1 world coordinate	(5, 15, 35)
LED 2 world coordinate	(3, 19, 35)
LED 3 world coordinate	(8, 12, 35)
LED 4 world coordinate	(9, 11, 35)
Tilt shift on the *x*-axis	0${}^{\circ }$–60${}^{\circ }$
Tilt shift on the *y*-axis	0${}^{\circ }$–60${}^{\circ }$

**Table 2:** Positioning errors regarding AS accuracy.

Angle Errors (degree)	Distance Estimation in Equation (3) (m)	Positioning Error in Equation (4) (m)
0.1	$5.89\times 10^{-9}$	$8.46\times 10^{-11}$
0.3	$4.61\times 10^{-9}$	$9.68\times 10^{-10}$
0.5	$1.97\times 10^{-9}$	$7.16\times 10^{-11}$
1	$2.78\times 10^{-9}$	$3.26\times 10^{-10}$
1.5	$5.11\times 10^{-5}$	$9.65\times 10^{-8}$
2	$3.76\times 10^{-5}$	$4.56\times 10^{-7}$

**Table 3:** VLC-based positioning algorithms using IS.

Name	Reciever Base	Methods Used	Accuracy	Noise Reduction	Orientation
Yoshino [12]	IS	collinearity condition	7 cm	no	arbitrary
Nakazawa [13]	IS	collinearity condition	10 cm	no	arbitrary
Rahman [14]	2 × IS	two steps	15 cm	no	paralel
Kim [15]	2 × IS	two steps	85 cm	no	paralel
Proposed method	IS + AS	two steps	10 cm	yes	arbitrary
